# Characterising Heatwave Responses and Climate Driver Impacts Using Multicollinearity-Controlled Generalised Linear Mixed Models in Urban and Forest Trees (2018–2023)

**DOI:** 10.1007/s41748-025-00648-5

**Published:** 2025-05-15

**Authors:** Ramla Khan, Philip Wheeler, David Gowing

**Affiliations:** School of Earth, Environment and Ecosystem Sciences, The Open University, Milton Keynes, MK7 6AA UK

**Keywords:** Climate variables, Urban forestry, Deciduous forest, Satellite data, Heatwave events, Multicollinearity, Time-lag, Spatial maps

## Abstract

Climate change is intensifying stressors on tree ecosystems, creating an urgent need to understand how climate variables influence vegetation health. This study investigated the influence of leaf temperature, wind speed, surface pressure, and moisture levels on tree health in a deciduous forest at Aspley Heath and an urban area in Milton Keynes, UK, using the normalised difference vegetation index (NDVI) as a vegetation health indicator. Satellite data from 2018 to 2023 revealed multiple heatwave events, with leaf temperatures surpassing the critical thresholds of 38 °C and 42 °C, as determined through controlled experiments. Generalised linear mixed models (GLMMs) demonstrated strong interactions between climate variables and vegetation health. Increased leaf temperature was positively associated with NDVI in both ecosystems, though urban trees showed a weaker response (β = 0.32, 95% CI: 0.16–0.48, p < 0.001) compared to forest trees (β = 0.45, 95% CI: 0.35–0.55, p < 0.001), suggesting urban heat island effects limit thermal benefits. Since all predictors were standardised, these coefficients indicate that a 3.82 °C increase in leaf temperature corresponds to a 0.32 NDVI increase in urban areas and a 0.45 increase in forests, reflecting meaningful ecological responses to moderate warming. Wind speed had a consistently negative impact on NDVI, with stronger effects in urban areas **(**β = − 0.21, 95% CI: − 0.41 to − 0.02, p = 0.035) than in forests (β = − 0.17, 95% CI: − 0.29 to − 0.05, p = 0.004). Surface pressure showed positive associations with vegetation health (urban: β = 0.17, 95% CI: 0.03–0.31, p = 0.02; forest: β = 0.14, 95% CI: 0.04–0.24 p = 0.005), while moisture availability emerged as a critical factor, with forest trees displaying a stronger response **(**β = 0.39, 95% CI: 0.29–0.49, p < 0.001) compared to urban trees (β = 0.30, 95% CI: 0.20–0.40, p < 0.001). Additionally, time-lag analysis revealed that leaf temperature had both an immediate effect on NDVI (lag 0: β = 0.64, p < 0.001) and a smaller delayed influence the following day (lag + 1: β = 0.18, p < 0.001) on NDVI, highlighting short-term vegetation sensitivity to thermal stress. These findings highlight how urban and forest trees differ in their resilience to climate variables, emphasising the need for targeted environmental management strategies to mitigate climate-induced stressors and protect tree ecosystems.

**Graphical Abstract:**

The graphical abstract provides a concise visual summary of the study, illustrating how climate variables impact tree health in contrasting urban and forest environments. Using satellite-derived NDVI data and Generalised Linear Mixed Models (GLMMs), the abstract displays the standardised effect sizes (β-values) for key leaf temperature, wind speed, surface pressure, and moisture availability predictors. Each climate variable is clearly labelled alongside its corresponding β coefficient to improve interpretability. Leaf temperature positively influenced vegetation health in both ecosystems, with a stronger effect in forests (β = 0.45) than in urban areas (β = 0.32). Similarly, moisture availability showed a more substantial effect in forests (β = 0.39) compared to urban areas (β = 0.30). Wind speed negatively affected tree health, with a more pronounced impact in urban environments (β = − 0.21) than in forests (β = − 0.17). Surface pressure showed a smaller but positive influence in both settings (urban: β = 0.17; forest: β = 0.14). A colour gradient background from cool blue-green on the forest side to warm orange-red on the urban side, as well as the blue and red thermometers respectively, represents the cooler environment in the forest compared to the warmer urban area. A thermometer icon with the warming planet next to the heatwave graph and spatial maps visually highlights the extreme heat events.

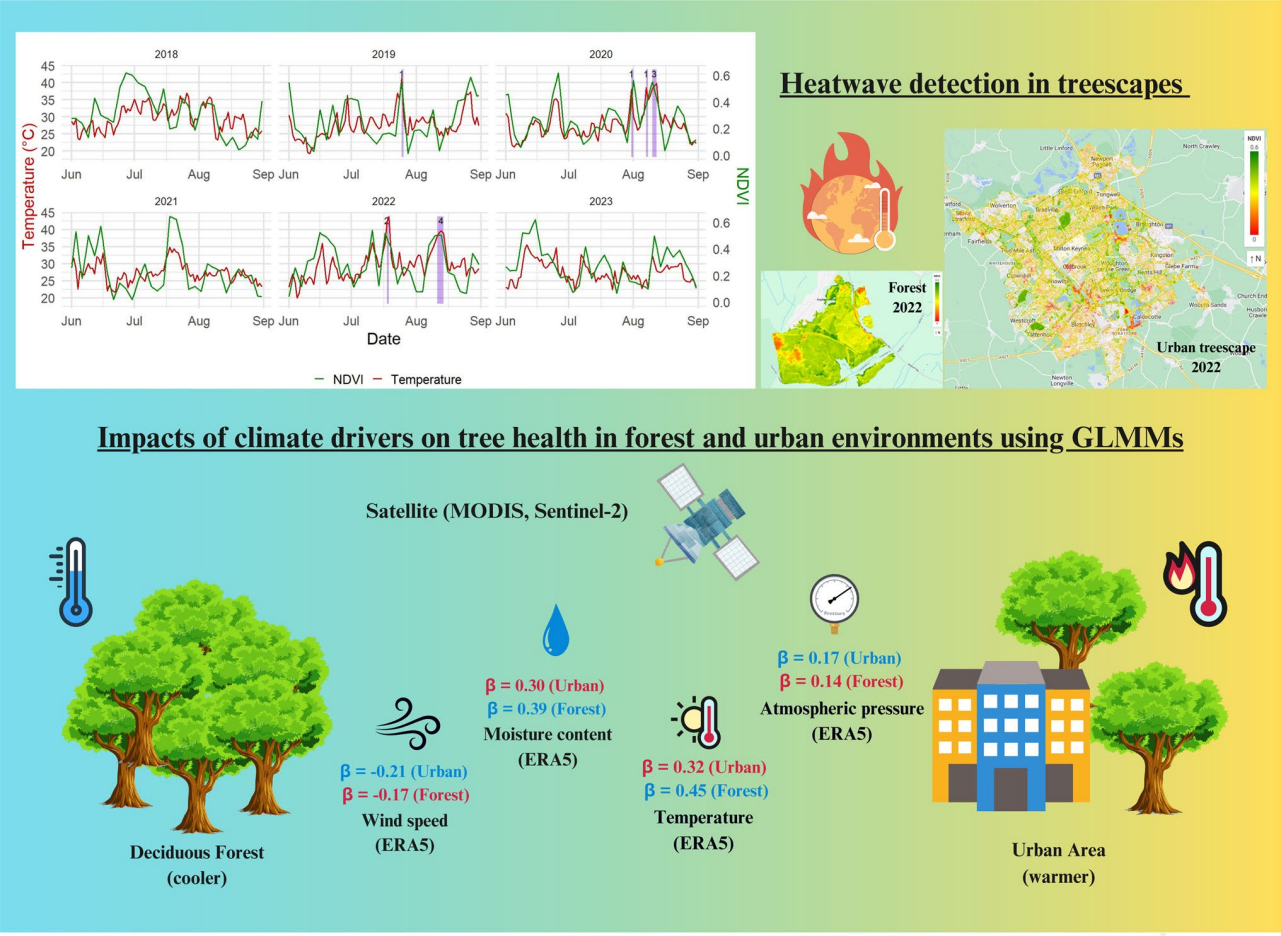

## Introduction

Extreme temperature events have increased across the globe both in frequency and intensity since the mid-twentieth century (IPCC 2023), and this global trend is also mirrored in national and regional climates. During the decade 2012–2021, the United Kingdom’s (UK’s) average temperature was 1 °C higher than the 1961–1990 baseline, exceeding the global surface temperature increase of 0.8 °C over the same period (Dessai et al. 2024). Heatwaves, characterised by prolonged periods of high temperature and humidity, exert significant stress on ecosystems (Ahrens et al. 2021; Cochavi et al. 2020; Marchin et al. 2022; Murakami et al. 2021). In the United Kingdom, the Met Office defines heatwaves based on region-specific temperature thresholds maintained for at least three consecutive days, with values ranging from 25 °C in northern regions to 28 °C in London (Kendon et al. 2022). However, this definition is tailored to human health and does not reflect thresholds relevant for vegetation, particularly trees, underscoring the need for plant-specific thresholds. Our study defined critical temperature thresholds for urban trees based on controlled experiments conducted on the university campus in Milton Keynes, UK. These thresholds derived had an initial critical temperature (T_crit_) of 38 °C during direct heat exposure, and a secondary threshold of 42 °C after maintaining leaf samples under hydrated conditions for 24 h (Khan et al. 2025b). The critical temperature is the temperature beyond which significant declines in photosynthetic efficiency (F_v_/F_m_) occur, indicating the onset of thermal stress (Krause et al. 2010; Perez and Feeley 2020; Schreiber and Berry 1977).

Trees are widely regarded as a cost-effective, nature-based solution for mitigating rising temperatures (Aram et al. 2019). However, as climate change intensifies, the tree ecosystem faces mounting challenges. They are increasingly vulnerable to extreme heat events, which accelerate senescence and increase mortality (Moser-Reischl et al. 2018). Beyond climate change, land-use changes, such as urbanisation and deforestation, alter trees’ exposure to environmental stressors (Feeley et al. 2020). The urban heat island (UHI) effect, where urban areas experience higher temperatures than surrounding rural regions, intensifies climate-induced stress on vegetation (Cheela et al. 2021; Moser-Reischl et al. 2018). However, lower-density residential developments can contribute more to radiant heat accumulation than high-density areas due to increased surface exposure and fragmented tree cover (Stone and Rodgers 2001).

Urban trees and green spaces counteract the UHI effects through shade provision and transpiration cooling (Armson et al. 2012). Strategic tree planting in high-density areas has been shown to reduce daytime heat accumulation and improve microclimate conditions (Tan et al. 2021; Wang and Akbari 2016). Moreover, greater tree species diversity enhances cooling effects, with biodiversity playing a crucial role in mitigating urban heat stress (Wang et al. 2021).

Remote sensing offers an efficient way to monitor vegetation dynamics and assess the impacts of climate stressors on trees. Satellite imagery in particular offers cost-effective and extensive spatial coverage, making it valuable for studying land cover changes and ecological responses to environmental stressors (Prudente et al. 2020; Willis 2015; Ardila et al. 2011; Baldeck et al. 2015). These large-scale observations complement field-based observations by providing temporally consistent data across broad spatial scales (Berra et al. 2019). A widely used remote sensing metric, the normalised difference vegetation index (NDVI), is derived from red and near-infrared spectral bands and serves as a key indicator of vegetation health and productivity (Khan and Gilani 2021; Gu et al. 2007).

To effectively analyse the relationship between remotely sensed vegetation indices and climate variables, robust statistical approaches are necessary. Generalised linear mixed models (GLMMs) are particularly well-suited for analysing climate-vegetation interactions as they can handle complex relationships and hierarchical data structures (Bates et al. 2015; Bolker et al. 2009; Harrison et al. 2018). In plant sciences, researchers frequently use GLMMs to separate the random and fixed effects of the response variable from the independent variables (Goñas et al. 2022; Patra et al. 2024). However, multicollinearity (high correlation between predictor variables) can bias model estimates and obscure the true effects response variable (Dormann et al. 2013; Shrestha 2020; Singh et al. 2023; Willan and Watts 1978; Zhang and Xiao-Ping Zhou 2020). This issue is particularly relevant in climate studies, where variables such as humidity and transpiration often exhibit strong interdependence. To ensure robust statistical inference, addressing multicollinearity is critical when modelling vegetation responses to climate stressors (Graham 2003; Qasim et al. 2020).

Time lag is another issue commonly faced in time series climate studies. A lagged effect refers to the delayed influence of one variable on another, with a lag of one or two indicating that the impact manifests in the subsequent or second period, respectively (Rijnhart et al. 2022). Understanding time lags is essential for predicting plant behaviour under changing climatic conditions and for developing strategies to mitigate the impacts of climate change on plant ecosystems (Masuda et al. 2022; Zhu et al. 2023).

Trees in urban environments experience heightened stress due to the compounded effects of heat waves, pollution, and altered hydrological cycles compared to those in natural forests. By directly comparing urban and forest responses to climate drivers using satellite-derived NDVI, this study offers new insights into the resilience of tree ecosystems under extreme conditions with direct relevance for urban forestry and climate adaptation strategies. By quantifying vegetation responses across different environments, this research provides novel insights into the differential resilience of trees under increasing climatic pressures, with implications for urban forestry management and climate adaptation strategies.

This study provides a novel approach by (1) defining physiologically relevant heatwave thresholds for urban trees using controlled experiments, rather than relying solely on meteorological definitions; (2) exploring the temporal dynamics of vegetation responses through lagged correlations between leaf temperature and NDVI; and (3) mapping spatial NDVI changes before and after identified heatwave events to visualise ecological impacts at high resolution. Furthermore, the study controls for multicollinearity among climate predictors to ensure robust statistical inference in a generalised linear mixed model, allowing for direct comparison between urban and forest tree responses to climate stressors. By combining physiological, temporal, and spatial perspectives, this research offers an integrated framework for assessing tree vulnerability in the context of intensifying climate extremes.

## Methods

This study employed a multi-step approach combining satellite remote sensing, climate datasets, and statistical modelling to investigate the heatwave responses of urban and forest vegetation from 2018 to 2023 in the summer months (June to August). We extracted NDVI data using MODIS (for temporal analysis) and Sentinel-2 (for spatial mapping), alongside climate variables for modelling.

Satellite data processing was carried out using the cloud-based platform Google Earth Engine (GEE). Statistical analyses and modelling were conducted in the R programming language, while spatial mapping of the study sites was performed using QGIS. Climate and vegetation health datasets were obtained from the GEE data repository.

Tree cover information was derived from land cover data at a 10 m resolution, published by the UK Centre for Ecology and Hydrology (UKCEH) (UKCEH 2022), which provides detailed classifications of vegetation types across the UK in 21 classes. To enhance the accuracy of the urban tree cover classification and minimise misclassification errors, we incorporated high-resolution infrastructure datasets from the Ordnance Survey (OS 2025), including building footprints, water bodies, road networks, and railway lines. These OS layers were used to generate exclusion masks that were applied to the initial tree cover data. By removing pixels that spatially overlapped with non-vegetated surfaces such as built infrastructure and open water bodies, we ensured that only genuine vegetative cover was retained in the final urban tree mask. This preprocessing step was critical for improving the reliability of NDVI extraction and subsequent modelling efforts.

## Study Sites

### Urban Area

**Fig. 1:**
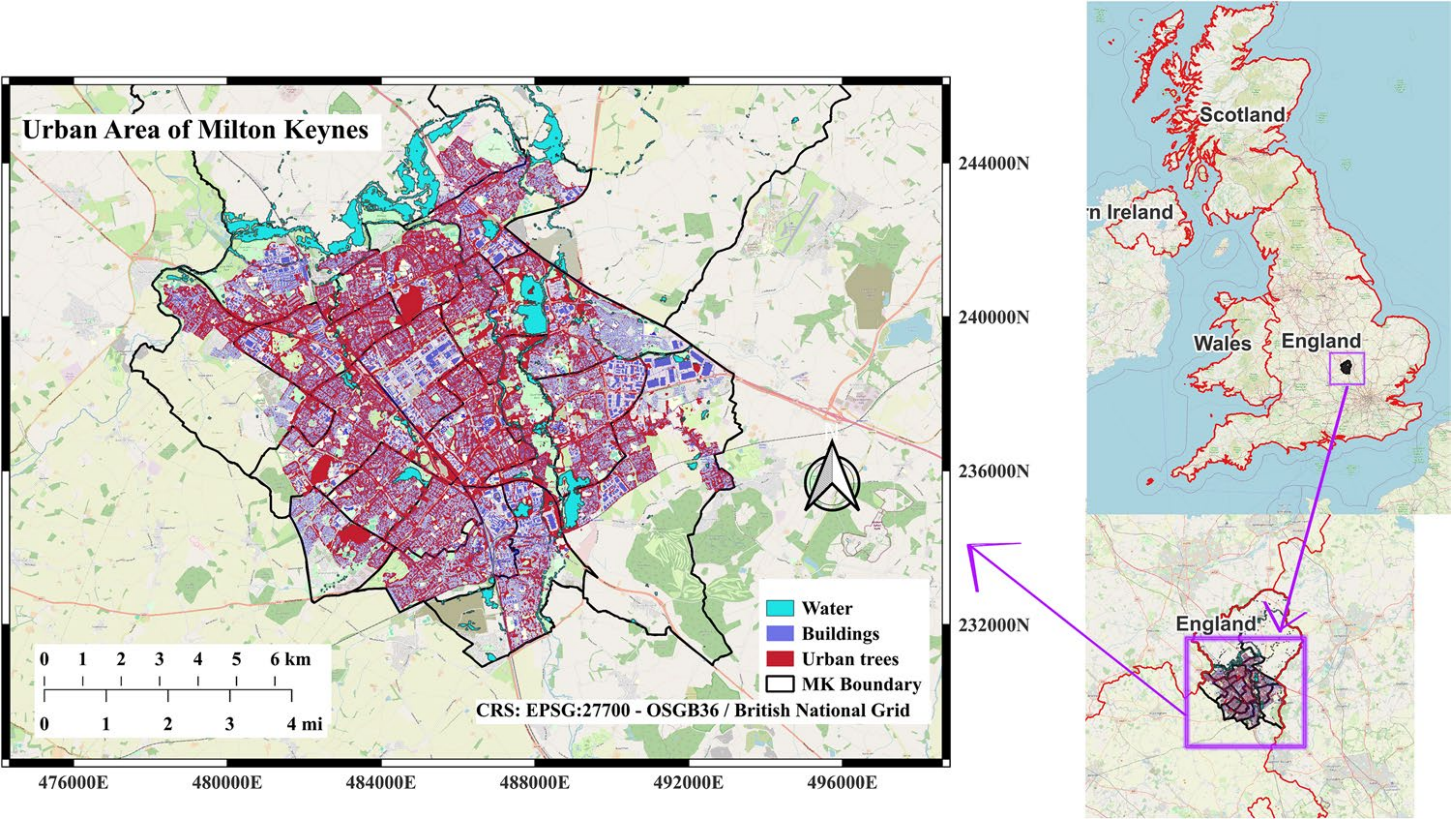
The urban area of Milton Keynes

Milton Keynes (MK) (Fig. 1), a planned city in Buckinghamshire, England, was designed in 1967 with a distinctive grid road layout and extensive green spaces. The city incorporates innovative urban planning features, including wide boulevards and roundabouts, which contribute to its unique urban landscape. The coordinates for Milton Keynes are roughly SP 822 402. The city is situated about 80 kms (50 miles) north of London and is part of the Southeast of England. Its location places it in a relatively flat, lowland area with a mix of urban and rural landscapes, including extensive green spaces and parks, making it a unique site for studying urban tree physiology.

Milton Keynes has a temperate oceanic climate, with moderate seasonal variation in temperature distributed fairly evenly throughout the year. The warm season typically spans from June to August, with July being the hottest month, reaching average highs of around 22 °C. While historically mild, the region has experienced increasingly frequent heat extremes in recent years, with temperatures occasionally exceeding 30 °C during summer heatwaves (Weather Spark 2025).

### Deciduous Forest

**Fig. 2:**
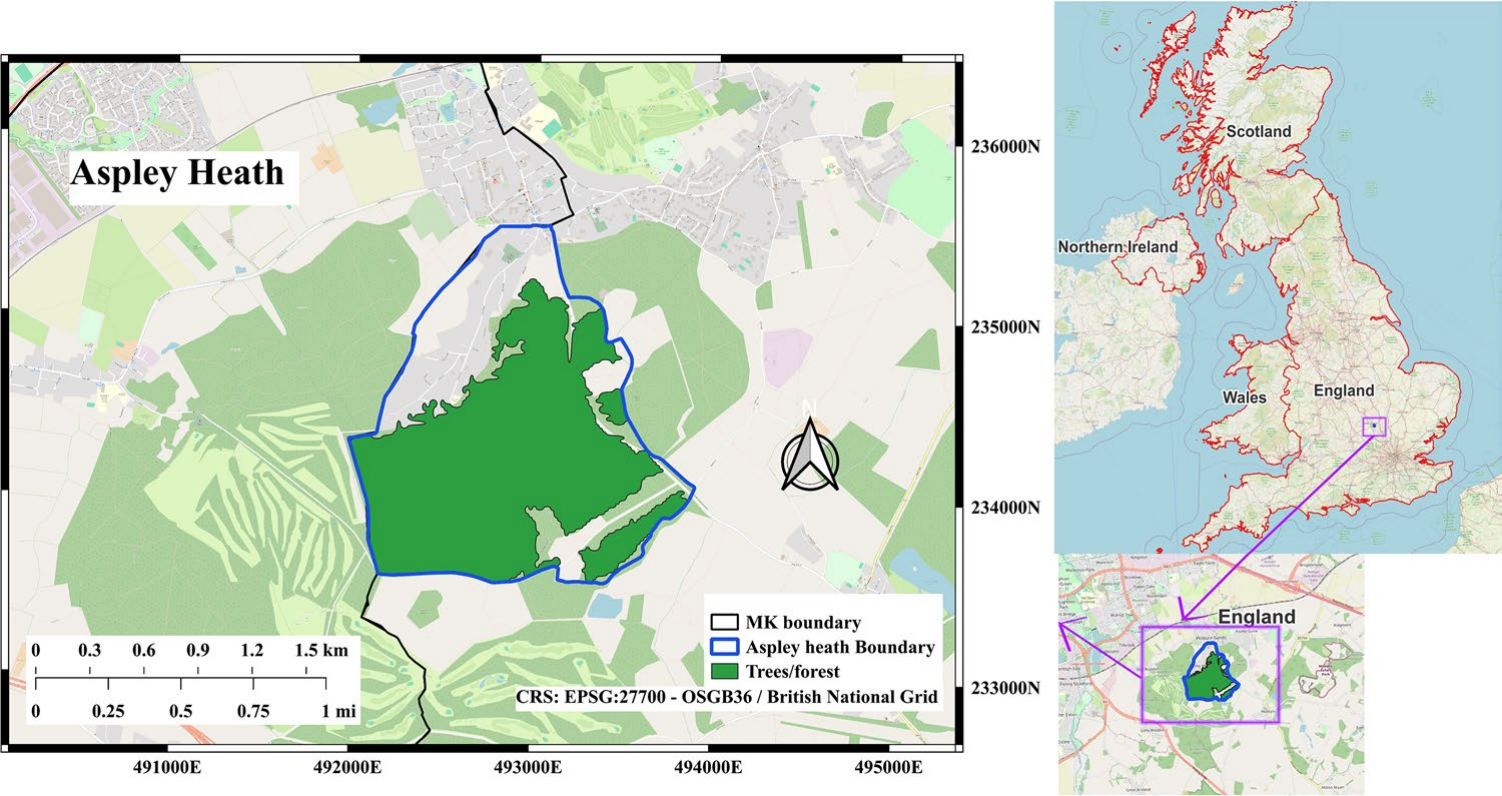
The forest of Aspley Heath

To assess how urban and natural forest trees respond to environmental stressors, a deciduous forest in Aspley Heath (Fig. 2) was selected as a natural reference site. By selecting a forest with similar species composition and phenological patterns, this study minimised confounding effects related to differences in tree life cycles.

The geographic proximity of Aspley Heath to Milton Keynes ensured that trees in both locations experience comparable regional climatic conditions and seasonal variability. This allowed for a more controlled assessment of urbanisation effects on tree physiology, particularly in response to heat stress.

### Datasets

In this study, we focused exclusively on summer seasons (June through August) in years 2018 to 2023, when thermal stress on vegetation is most pronounced.

### Vegetation Health

NDVI time-series data were obtained from the MOD09GQ v6.1 MODIS Surface Reflectance product, which provides 250 m resolution daily imagery (Roger et al. 2020). The product's quality control (QC) band was used to mask cloud-contaminated pixels, ensuring that only high-quality observations were included in the analysis. Specifically, we retained only pixels with clear sky conditions based on the QC flags. NDVI was computed from the near-infrared (band 2, 841–876 nm) and red (band 1, 620–670 nm) bands using the standard formula: NDVI = (NIR − RED)/(NIR + RED). This approach enabled us to track vegetation health during summer heatwave periods, providing a critical indicator of ecosystem response to thermal stress. 387 images after pre-processing were obtained for the study.

For the spatial maps of NDVI, Sentinel-2 multispectral Instrument, Level-1c (ESA 2015) satellite data was initially filtered to exclude images with more than 30% cloud cover using the CLOUDY_PIXEL_PERCENTAGE metadata property. Subsequently, both dense and cirrus clouds were removed to produce clearer imagery free from shadows and cloud contamination using the QA60 band, where bits 10 and 11 represent dense clouds and cirrus clouds, respectively. A binary mask was created where both these bits were set to zero, indicating clear conditions.

Dense clouds are sometimes misclassified as snow cover, so the B11 and B12 bands in the SWIR spectrum were incorporated into the analysis. To differentiate between ice/snow at high altitudes and dense clouds, the reflectance of B10 was analysed in combination with other bands to improve discrimination between these features. The mask for cirrus clouds identifies pixels as most likely being cirrus clouds when they have a high reflectance value in the B10 and a low reflectance value in the blue band or B2. After cloud masking, NDVI was calculated using the normalised difference between the NIR (B8) and Red (B4) bands. The final NDVI maps were produced at Sentinel-2's native 10 m resolution from the 29 images obtained after pre-processing.

### Climate Data

Climate variables were extracted from the ECMWF/ERA5-Land Daily Aggregated (DAILY_AGGR) dataset, a high-resolution (0.1° × 0.1°, approximately 9 km) reanalysis product from the Copernicus Climate Data Store (Sabater 2019). The selected climate variables included humidity, maximum air temperature, wind speed, surface pressure, and transpiration rate. These variables were chosen due to their relevance in assessing heat stress and moisture availability, which are key factors influencing tree physiological responses. Data were extracted for summer months (June–August) to align with the NDVI analysis period.

**Fig. 3:**
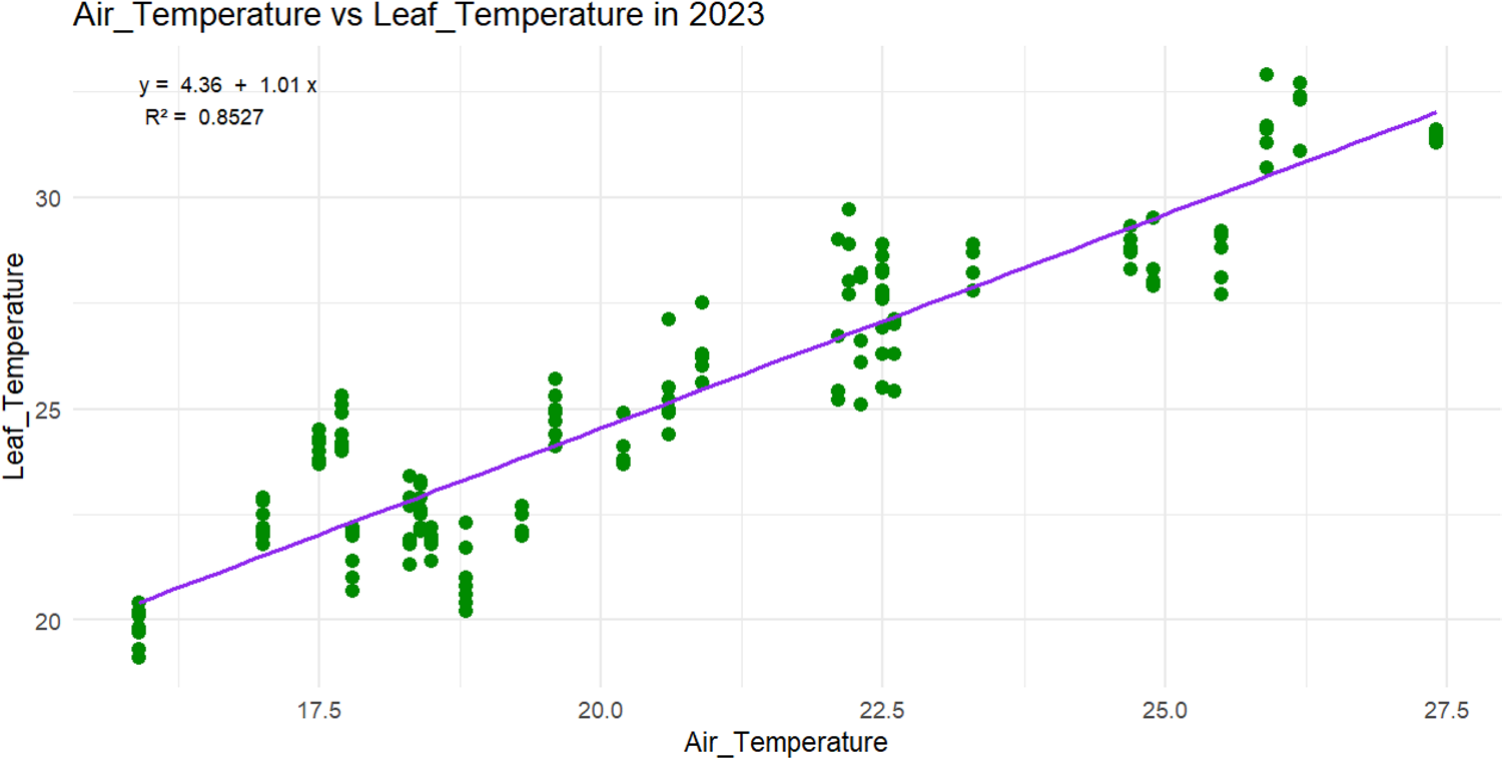
Leaf temperatures vs air temperature regression model

Leaf temperature was derived directly from the air temperature of the ERA5 dataset through a regression model (Fig. 3) by using the leaf temperature computed in another experiment by the researchers during season 2023 summer season as input (Khan et al. 2025a). Leaf temperature was considered a more physiologically relevant metric than solar radiation, as it reflected the net energy balance of the leaf and integrated multiple environmental drivers, including solar radiation, ambient temperature, wind speed, humidity, and transpiration rate. By including variables such as humidity, transpiration, and air pressure, we accounted for the plant’s evaporative environment, thereby capturing the indirect effects of solar radiation on physiological stress. Our final model employed biologically meaningful predictors, avoiding redundancy while still representing the key environmental factors influencing plant physiological responses.

The slope of the regression line was estimated to be 1.01, indicating a near-linear relationship between air and leaf temperature. The p-value for this slope was < 0.001, confirming that the relationship is statistically significant. Additionally, the intercept of the model was 4.36, meaning that leaf temperatures were typically higher than air temperatures by more than 4 °C.

### Statistical Analysis

All variables were standardised to reduce the influence of outliers before fitting the GLMM. Missing values (NA), which resulted from unsuitable images filtered by the MODIS NDVI quality control band, were interpolated using data from the preceding and following time points.

### Detection of Treescape’s Heatwaves

This heatwave definition was derived from a previous controlled study we conducted on urban trees on-site. In the experiment, we used common urban tree species found in the UK and Milton Keynes as test subjects and performed controlled lab experiments to derive the threshold critical temperature (T_crit_) (Khan et al. 2025b).

Table 1 presents the critical temperature (T_crit_) thresholds used in this study, including their upper and lower confidence intervals. The heatwave definition is based on the average T_crit_ values obtained from three tests conducted on the day of heat exposure and the subsequent 24-h hydration period.

**Table 1:** Heatwave threshold for urban trees from controlled experiments

Tests	T_crit_	CI_Lower	CI_Upper
T1	40.47	38	44
T2	36.88	34	39
T3	35.78	29	41
T1_24 hr	42.28	41	45
T2_24 hr	43.03	41	45
T3_24 hr	43.05	41	45

### Time Lag Detection

Potential lag structures were initially identified using cross-correlation analysis using the CCF function in R (Brockwell and Davis 1991). Time-lagged predictors corresponding to significant peaks were then included in the GLMM, and their statistical significance was evaluated based on the associated p-values.

### Multicollinearity Detection

Multicollinearity was assessed using the variance inflation factor (VIF)**,** which quantifies the degree of correlation between predictor variables (Shrestha 2020; Vatcheva et al. 2016). VIF values exceeding 5 were considered indicative of moderate collinearity, while values above 10 suggested severe collinearity requiring corrective measures.

### Modelling the Data

To analyse the relationship between climate drivers and vegetation response, generalised linear mixed models (GLMMS) were used. These models allow for the inclusion of both fixed and random effects, making them well-suited for handling repeated measures and hierarchical data structures (Bolker et al. 2009; Harrison et al. 2018; Patra et al. 2024). The lme4 package in R was used to fit the models (Bates et al. 2015).

GLMMS account for seasonal variations and autocorrelation in ecological time series data, making them particularly useful for detecting patterns in NDVI fluctuations under different climate conditions. By incorporating random effects, these models helped control for unmeasured dependencies in the dataset.

To investigate the impact of heat-induced stress on vegetation, we specifically focused on the summer season, which is most pertinent for assessing the effects of heat waves. Given the variability in the timing of extreme heat events in Milton Keynes, UK (Dessai et al. 2024), we opted to group the data by month-year rather than fixed seasonal blocks. This approach enabled us to capture the interannual variability in climatic conditions and vegetation responses, particularly in terms of NDVI. By adopting this strategy, we were able to better capture variations in heat stress across different years, thus ensuring that patterns were not masked by rigid seasonal definitions.

## Results

### Treescape’s Heatwaves

**Fig. 4:**
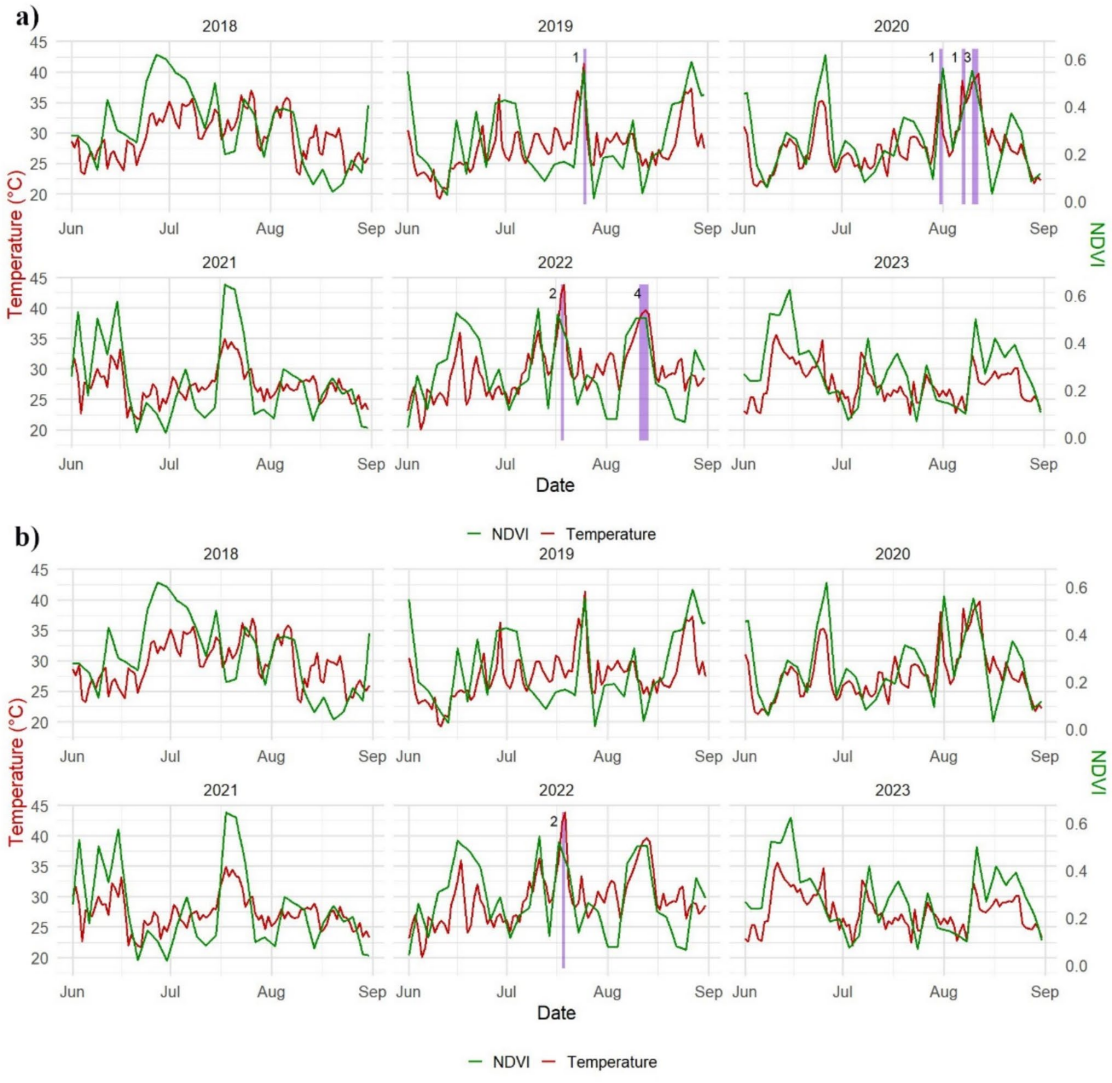
Heatwave events in the urban area of Milton Keynes with leaf temperature threshold of: **a** T_crit_ > 38 °C, **b** T_crit_ > 42 °C

Leaf temperature analysis identified multiple heat stress events between 2018 and 2022, based on the 38 °C threshold (Fig. 4a, Table 2). The heat events included a single day of extreme heat in July 2019, multiple short heat events in 2020 (July 31 st, August 7 th, and 3 days from August 10–12), and two heat events in 2022 (July 18–19 and August 11–14).

**Table 2:** Heat stress events in the urban area of Milton Keynes when T_crit_ > 38 °C

Year	Start date	End date	Duration
2019	2019–07–25	2019–07–25	1
2020	2020–07–31	2020–07–31	1
2020	2020–08–07	2020–08–07	1
2020	2020–08–10	2020–08–12	3
2022	2022–07–18	2022–07–19	2
2022	2022–08–11	2022–08–14	4

This approach offered a tree-specific definition of heatwaves, extending beyond conventional meteorological criteria to focus on the actual physiological impact on urban vegetation.

Applying the higher 42 °C threshold resulted in fewer recorded heat events (Fig. 4b). Notably, only in July 2022 did leaf temperatures reach this critical point, with temperatures peaking at 42.9 °C in the Urban area of Milton Keynes.

**Fig. 5:**
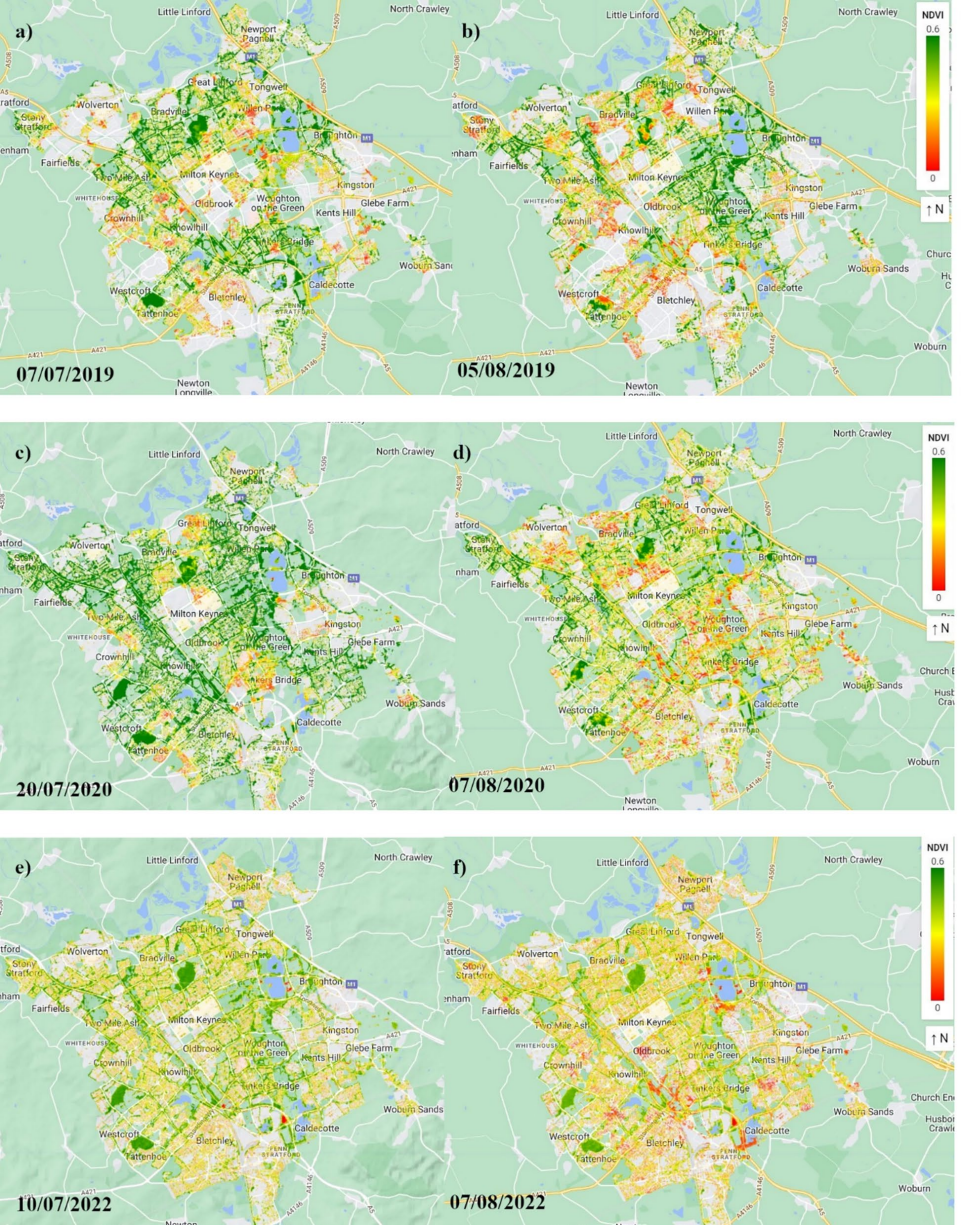
NDVI before and after heatwave events in Milton Keynes urban area: **a**, **b** 2019, **c**, **d** 2020, and **e**, **f** 2022. Left panels (**a**, **c**, **e**) show NDVI measurements before heatwave events, and right panels (**b**, **d**, **f**) show NDVI measurements after heatwave events. Green colours indicate high vegetation health, while yellow and red indicate progressively lower vegetation health

Figure 5 shows how the tree cover looked before and after heatwave events described in Table 1 using the NDVI data derived from Sentinel-2 in high resolution. There was no suitable image available for the selected area that showed aftereffects of a heatwave with start date 2022–08–11, hence no spatial map was included in Fig. 5.

**Fig. 6:**
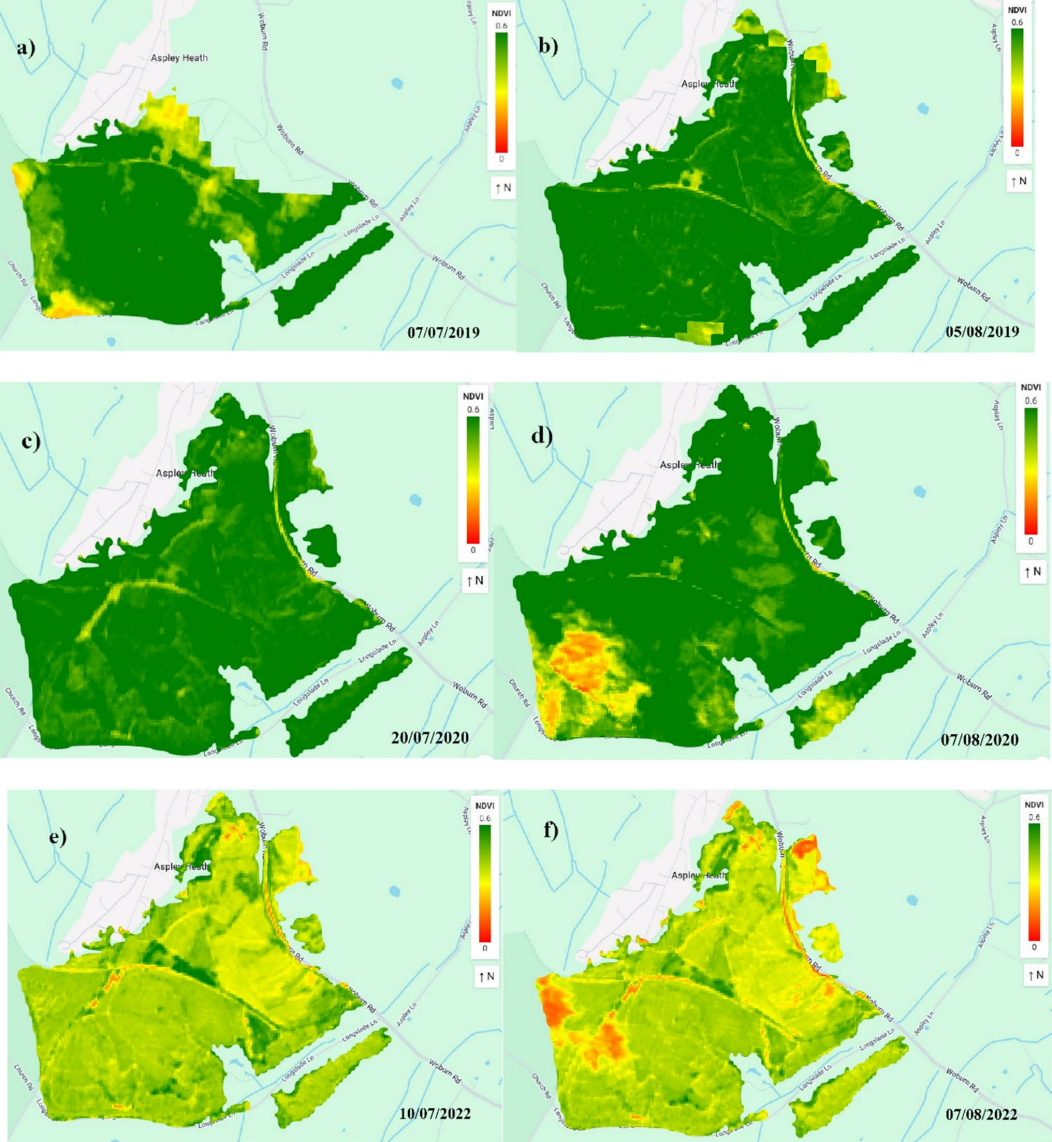
NDVI before and after heatwave events in Aspley Heath Forest: **a**, **b** 2019, **c**, **d** 2020, and **e**, **f** 2022. Left panels (**a**, **c**, **e**) show NDVI measurements before heatwave events, and right panels (**b**, **d**, **f**) show NDVI measurements after heatwave events. Green colours indicate high vegetation health, while yellow and red indicate progressively lower vegetation health

The threshold of heatwave was specified for the urban area, but to make a comparison with the forest area, we generated spatial maps of NDVI for the same dates and range in Fig. 6.

### Time Lag Between NDVI and Leaf Temperature

**Fig. 7:**
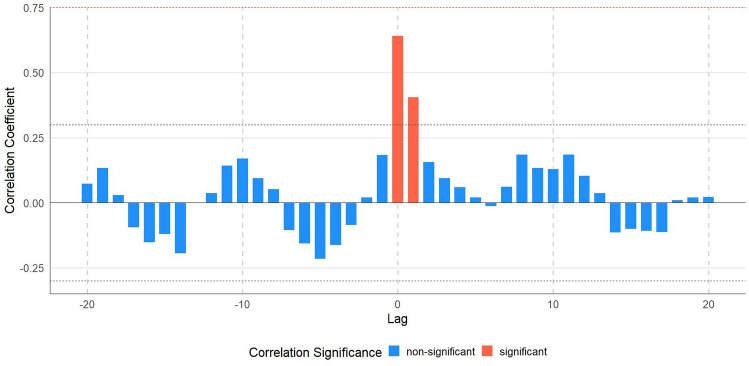
Cross-correlation function (CCF) between leaf temperature and NDVI for the urban area

To examine potential time lags between NDVI and climate variables, we applied the cross-correlation function (CCF) in R. We did not observe any significant lag between NDVI and other climate variables except the leaf temperature. Figure 7 indicates a significant correlation at lag 0, representing the immediate relationship between leaf temperature and NDVI on the same day. At lag + 1, we observed a delayed effect, where the previous day's leaf temperature influenced the NDVI.

In Table 3, a strong positive correlation at lag 0 suggests that the NDVI is closely associated with leaf temperature on the same day. The smaller but still significant correlation at lag + 1 suggests a delayed response in NDVI due to previous-day leaf temperature, highlighting the temporal dynamics of vegetative response to thermal conditions in the urban environment.

**Table 3:** Lagged effect between leaf temperature and NDVI in the urban area of Milton Keynes

Lag	Estimate (β)	p-value	R^2^
0	0.64	** < 0.001**	0.4
1	0.18	** < 0.001**	0.03

Bold font indicates p-values less than 0.05, considered statistically significant

### Multicollinearity Correction

Initial variable diagnostic tests revealed several collinearity issues that required correction before proceeding with the GLMM analysis. Leaf temperature exhibited high collinearity (VIF = 9.45), while humidity (VIF = 6.20) and transpiration rate (VIF = 6.07) showed moderate collinearity. Wind speed (VIF = 1.90) and surface pressure (VIF = 1.20) demonstrated acceptable low collinearity values.

To address the moderate collinearity between humidity and transpiration rate, representing the components of tree moisture stress, principal component analysis (PCA) was applied (Çankaya and Samet Eker 2019). This approach preserved the essential information while reducing redundancy between these highly correlated variables, creating a combined moisture index that better represented the overall moisture conditions experienced by trees.

Following PCA adjustment, all predictor variables exhibited low collinearity: leaf temperature (VIF = 1.06), surface pressure (VIF = 1.20), wind speed (VIF = 1.30), and the combined humidity-transpiration index (VIF = 1.12). These adjustments ensured that the GLMMs were free from significant collinearity, improving the reliability of the statistical estimates and allowing for clearer interpretation of individual climate variable effects.

### GLMM Validation and Fit

**Fig. 8:**
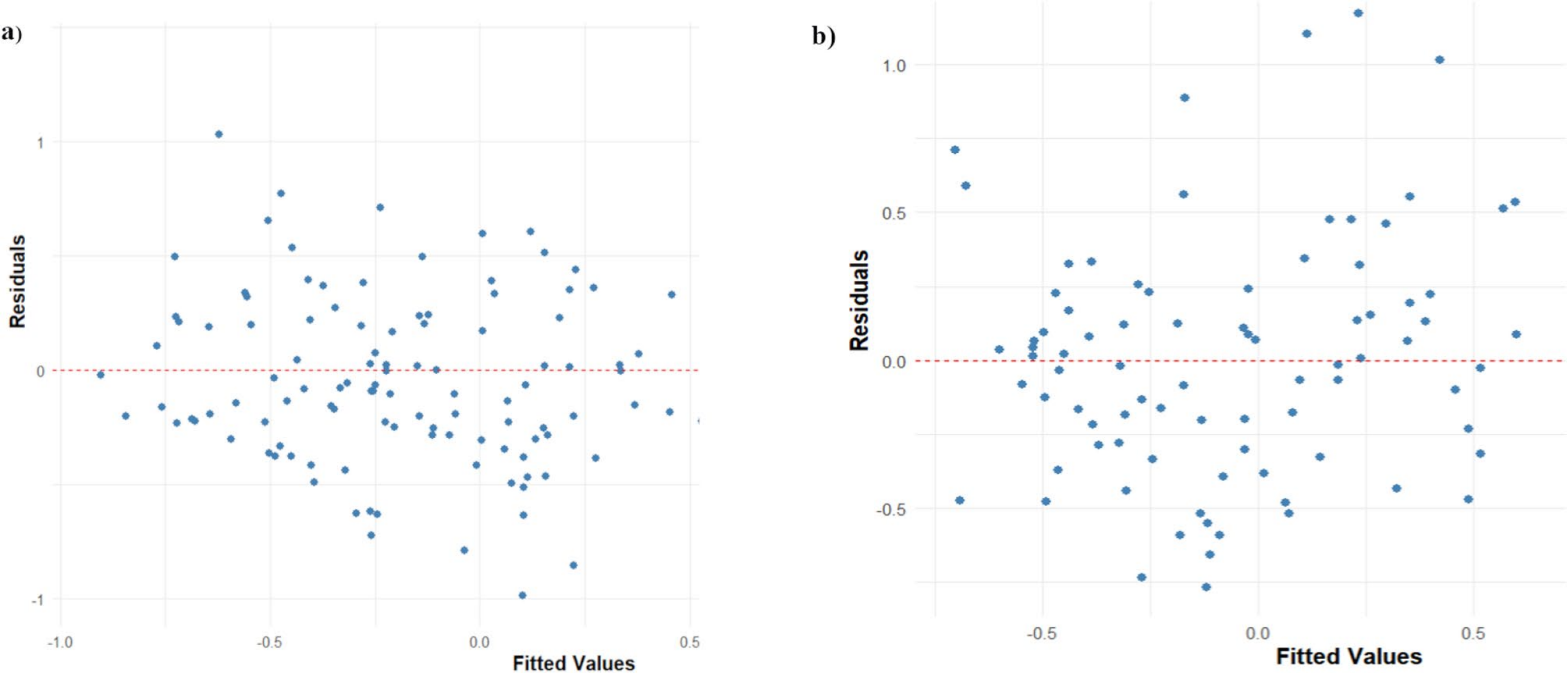
Residual vs fitted values plot of the GLMM: **a** Urban area, **b** for forest data

Model validation was performed through residual analysis to ensure the assumptions of the GLMMS were met. Figure 8 presents the residual versus fitted values plots for both the urban and forest models. The random distribution of residuals around zero with no discernible patterns indicated that the models adequately captured the relationships between the predictor variables and NDVI. This validation step confirmed the appropriateness of the GLMMs for analysing the climate-vegetation relationships in both ecosystems.

The models demonstrated good explanatory power, with the forest model explaining 53% of the variance in NDVI through fixed effects alone (marginal R^2^ = 0.53) and 63% when including both fixed and random effects (conditional R^2^ = 0.63). The urban model showed slightly lower but still substantial explanatory power, with fixed effects explaining 48.0% of the variance (marginal R^2^ = 0.48) and the combination of fixed and random effects explaining 56.9% (conditional R^2^ = 0.57). The intra-class correlation coefficients (ICC) indicated that approximately 20% of the total variance in forest NDVI and 17% in urban NDVI could be attributed to seasonal differences across years.

### Climate Variable Effects on Urban Tree Health

**Fig. 9:**
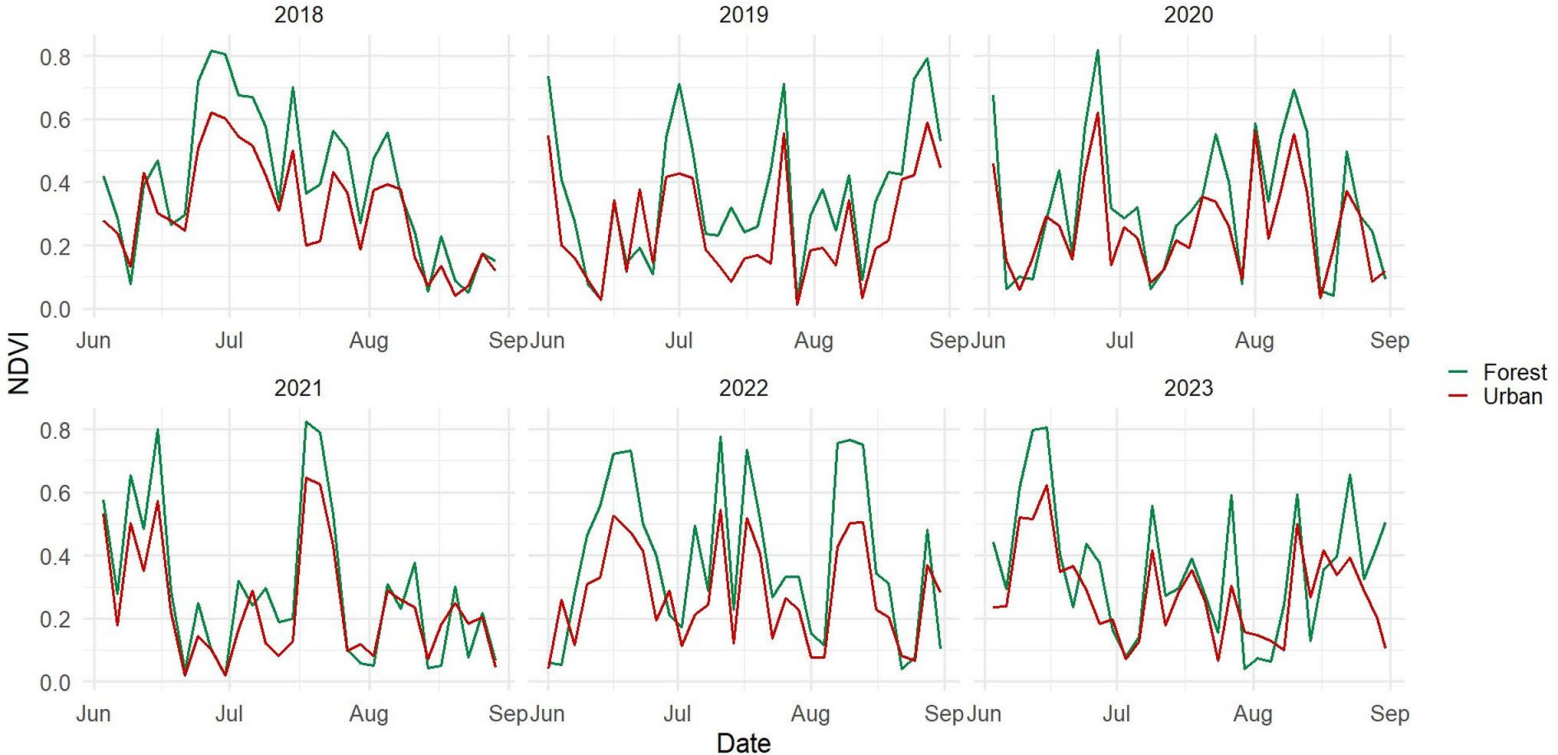
NDVI values from 2018 to 2023 in the urban area of Milton Keynes (red solid lines) and the deciduous forest of Aspley Heath (green solid lines)

Figure 9 illustrates the NDVI trends from 2018 to 2023 for both the urban area of Milton Keynes and the deciduous forest at Aspley Heath. The time series revealed seasonal variations in vegetation health, with higher NDVI values during peak growing seasons and notable year-to-year variability. The forest ecosystem consistently maintained higher NDVI values compared to the urban environment, reflecting greater vegetation density and canopy coverage. Particularly notable were the reduced NDVI values during the summer of 2022, which coincided with a significant heatwave and drought conditions across the UK, demonstrating the sensitivity of both ecosystems to extreme climate events.

The urban environment (Table 4) showed directional relationships between climate variables and NDVI, but with notable differences in effect magnitudes. Leaf temperature maintained a positive but reduced effect on urban vegetation health **(**β = 0.32, 95% CI: 0.16–0.48, p < 0.001) compared to the forest ecosystem, suggesting additional constraints from urban heat island effects that may dampen the beneficial aspects of warmer temperatures. Given that all predictors were standardised, this coefficient corresponds to an approximate increase of 0.32 in NDVI for every 3.82 °C rise in leaf temperature. This practical interpretation underscored that even moderate increases in temperature positively influenced the urban vegetation health.

**Table 4:** GLMM results for the urban area of Milton Keynes

Predictors	NDVI			95% CI (Lower–Upper)
	Estimates	Std. Error	p-value	
(Intercept)	0.00	0.07	0.96	− 0.14–0.14
Leaf temperature	0.32	0.08	** < 0.001**	0.16–0.48
Surface pressure	0.17	0.07	**0.02**	0.03–0.31
Wind speed	− 0.21	0.10	**0.03**	− 0.41 to − 0.02
Moisture index	0.30	0.05	** < 0.001**	0.20–0.40
*Random Effects*				
σ^2^	0.20			
τ_00_ _month_year_	0.04			
ICC	0.17			
N _month_year_	18			
Marginal R^2^/Conditional R^2^	0.48/0.57			

Bold font indicates p-values less than 0.05, considered statistically significant

The negative impact of wind speed was more pronounced in the urban setting **(**β = − 0.21, 95% CI: − 0.41 to − 0.02, p = 0.035), potentially due to altered wind patterns created by buildings and infrastructure that may exacerbate mechanical stress on urban trees. Surface pressure showed a slightly stronger positive effect in urban areas (β = 0.17, p = 0.021) compared to forests, while the moisture index had a reduced positive effect (β = 0.30, p < 0.001), indicating that urban trees may not fully benefit from available moisture due to impervious surfaces and altered drainage patterns.

### Climate Variable Effects on Forest Tree Health

In the deciduous forest ecosystem (Table 5), leaf temperature emerged as the strongest positive predictor of NDVI (β = 0.45, 95% CI: 0.35–0.55, p < 0.001), indicating that within the observed temperature range, warmer leaf temperatures were associated with enhanced photosynthetic activity and overall vegetation health. This suggests that a 3.82 °C increase in leaf temperature is associated with a 0.45 increase in NDVI, indicating enhanced vegetation health in response to warming, particularly in the forest ecosystem.

**Table 5:** GLMM results for the forest of Aspley Heath

Predictors	NDVI			95% CI (Lower–Upper)
	Estimates	Std. Error	p-value	
(Intercept)	0.01	0.07	0.907	− 0.13–0.15
Leaf temperature	0.45	0.05	** < 0.001**	0.35–0.55
Wind speed	− 0.17	0.06	**0.004**	− 0.29 to − 0.05
Surface pressure	0.14	0.05	**0.005**	0.04–0.24
Moisture index	0.39	0.05	** < 0.001**	0.29–0.49
*Random Effects*				
σ^2^	0.24			
τ_00_ _month_year_	0.06			
ICC	0.20			
N _month_year_	18			
Marginal R^2^/Conditional R^2^	0.53/0.63			

Bold font indicates p-values less than 0.05, considered statistically significant

The moisture index also showed a strong positive relationship with NDVI (β = 0.39, p < 0.001), highlighting the critical role of water availability in supporting forest productivity. Surface pressure exhibited a moderate positive effect on forest NDVI (β = 0.14, p = 0.005), suggesting that stable atmospheric conditions, typically associated with higher pressure systems, were beneficial for tree health. Wind speed showed a significant negative impact on forest NDVI (β = − 0.17, 95% CI: − 0.29 to − 0.05, p = 0.004), likely due to increased evapotranspiration and potential physical damage to leaves and branches during periods of stronger winds.

### Comparative Analysis of Urban and Forest Responses

**Fig. 10:**
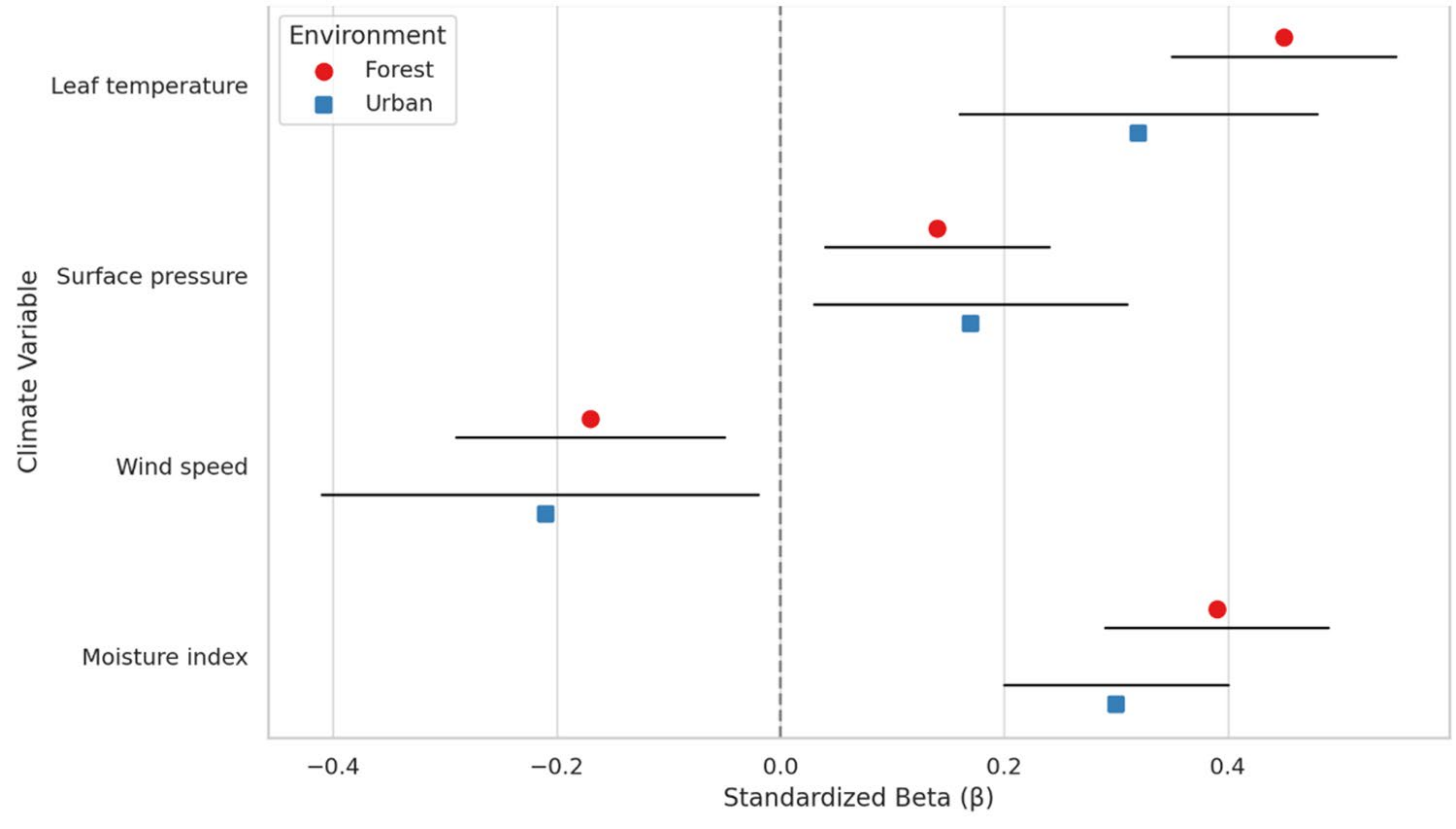
Standardised effect sizes (β) with 95% confidence intervals for climate variables in forest and urban environments

Figure 10 presents a comparative analysis of the standardised effect sizes and 95% confidence intervals for each climate variable across forest and urban environments. This visualisation highlights both similarities and key differences in how trees respond to climatic drivers depending on their environmental context.

The most notable differences were observed in the effects of leaf temperature and moisture availability. Forest trees exhibited a significantly stronger positive response to leaf temperature (β = 0.45, CI: 0.35–0.55) than urban trees (β = 0.32, CI: 0.16–0.48), suggesting that urban trees face additional constraints that limit their ability to benefit from favourable temperature conditions. Similarly, the influence of moisture availability was greater in forest environments (β = 0.39, CI: 0.29–0.49) compared to urban settings (β = 0.30, CI: 0.20–0.40), reflecting the compounded challenges posed by impervious surfaces, compacted soils, and altered hydrology in urban areas.

Wind speed exerted a more negative influence on urban vegetation (β = − 0.21, CI: − 0.41 to − 0.01) than on forest trees (β = − 0.17, CI: − 0.29 to − 0.05), potentially due to wind-tunnelling effects and increased mechanical stress caused by built infrastructure. In contrast, surface pressure had a modest positive effect in both environments, with a slightly stronger association observed in urban areas (urban: β = 0.17, CI: 0.03–0.31; forest: β = 0.14, CI: 0.04–0.24).

These comparative results indicate that while the direction of responses to climate variables remains broadly consistent across ecosystems, the magnitude and certainty of these responses vary. Urban trees generally exhibit dampened responses to beneficial variables (e.g., temperature, moisture) and exaggerated sensitivity to stressors (e.g., wind), underscoring the heightened physiological challenges of growing in urban environments. Including confidence intervals enhances the interpretation of these results, offering a more robust understanding of where effects are both statistically significant and ecologically meaningful.

## Discussion

Understanding how climatic variables affect different ecosystems is crucial for effective environmental management. This study examined a deciduous forest in Aspley Heath alongside the urban treescape of Milton Keynes, using satellite data to assess the impact of climate variables on the health of the treescape. Across the summers of 2018 to 2023, the analysis assessed the influence of leaf temperature, wind speed, surface pressure, and moisture levels influenced vegetation health represented by the NDVI.

The novelty of this study lies in its integration of physiological thresholds, spatial analysis, and time-series modelling to characterise vegetation responses to heat stress. Unlike previous work that applies generic or human-centric heatwave definitions, we employed treescape-specific thermal thresholds derived from controlled experiments, enabling more ecologically relevant identification of heat events. Additionally, by incorporating cross-correlation analysis, we identified immediate and delayed NDVI responses to leaf temperature, capturing short-term vegetation sensitivity. The spatial mapping of NDVI before and after heatwave events further contextualised these physiological responses, visually demonstrating the contrasting impacts in urban versus forest environments. Importantly, multicollinearity among predictors was addressed before model fitting, improving the interpretability of GLMM estimates and strengthening the reliability of inferences about climate drivers in both ecosystems.

The study revealed a strong positive relationship between leaf temperature and NDVI in both environments, with a greater effect size in the forest (β = 0.45, 95% CI: 0.35–0.55, p < 0.001) compared to urban areas **(**β = 0.32, 95% CI: 0.16–0.48, p < 0.001). This suggests that increasing leaf temperature generally supports photosynthetic activity and tree health, particularly in natural ecosystems where moisture availability is less constrained. Since the leaf temperature variable was standardised before modelling, the β values represent the effect of a 3.82 °C increase in leaf temperature. Accordingly, urban trees showed a 0.32 NDVI increase per 3.82 °C rise, while forest trees showed a 0.45 NDVI increase, highlighting a stronger thermal benefit in forest environments. However, the comparatively weaker response observed in urban environments highlights underlying stress factors highlights potential stress factors, such as the urban heat island (UHI) effect (Cheela et al. 2021; Marando et al. 2019; Marchin et al. 2022), which could limit the benefits of moderate temperature increases. The seasonal patterns in NDVI trends support this conclusion, as NDVI values were notably lower in the summer of 2022 due to extreme heat and drought conditions across the UK (Dessai et al. 2024). The heatwave analysis further confirmed this trend, with multiple heat events recorded between 2018 and 2022. In July 2022, leaf temperatures in the urban area of MK peaked at 42.9 °C, exceeding the critical temperature threshold and demonstrating the extent of heat stress urban trees endure. These findings suggest that while moderate temperature increases may promote growth, prolonged heat stress and associated moisture deficits could eventually reduce vegetation health, particularly in urban areas where impermeable surfaces exacerbate water scarcity (Armson et al. 2012; Marando et al. 2019).

Our time-lag analysis further supports this by demonstrating an immediate relationship between leaf temperature and NDVI (lag 0: β = 0.64, p < 0.001), as well as a secondary effect the next day (lag + 1: β = 0.18, p < 0.001). This suggests that tree health not only reacts instantly to thermal stress but may also continue to be affected by prior-day heat exposure. This temporal sensitivity was especially evident in the urban environment, where compounded stressors such as impermeable surfaces and wind exposure could prolong physiological recovery after heat waves. These short-term lags underline the importance of early warning systems and rapid intervention during extreme heat events (Masuda et al. 2022; Stone and Rodgers 2001).

These findings align with previous studies that show urban trees experience more extreme thermal conditions, leading to heat stress and reduced resilience compared to forest trees (Frank and Just 2020; Wei and Zhang 2019). While moderate temperature increases can benefit tree growth, extreme conditions could lead to thermal stress, reducing photosynthetic efficiency and increasing vulnerability to pests and diseases (Allen et al. 2015; Laćan and McBride 2008; Wi et al. 2020).

Wind may induce mechanical damage to leaves and branches, reducing overall leaf area and consequently lowering NDVI (Tadrist et al. 2018). Additionally, increased evapotranspiration rates due to wind can lead to water stress, particularly during periods of low moisture availability. These findings underscore the necessity for protective interventions, such as enhancing windbreak structures to mitigate the negative effects of wind on tree health (Wasko et al. 2020). In our research, wind speed negatively influenced NDVI in both environments, but the effect was stronger in urban areas (β = − 0.21, 95% CI: − 0.41 to − 0.02, p = 0.035) than in forests (β = − 0.17, 95% CI: − 0.29 to − 0.05, p = 0.004). This suggests that wind not only accelerates evapotranspiration but also exerts mechanical stress, particularly in urban settings where buildings and infrastructure can create wind tunnels that amplify these effects. The reduced canopy cover in urban areas can also contribute to greater exposure of individual trees to wind stress, leading to higher vulnerability to damage and desiccation. These findings have substantial implications for urban forestry policy and management. Strategies such as increasing tree density, selecting wind-resistant species, and improving soil moisture retention could mitigate wind-related stress in urban environments. In forest ecosystems, natural windbreaks and dense canopy structures likely provide some protection, reducing the extent of wind-induced stress on tree health.

Surface pressure was positively associated with NDVI in both urban (β = 0.17, 95% CI: 0.03–0.31, p = 0.021) and forest ecosystems (β = 0.14, 95% CI: 0.04–0.24, p = 0.005). This relationship suggests that stable atmospheric conditions, often linked to high-pressure systems, support vegetation health by reducing physiological stress and promoting favourable growing conditions. Higher surface pressure is generally associated with clear, dry weather, which can benefit photosynthesis if moisture levels are sufficient (Ambrose et al. 2016; Chellemi and Britton 1992; Knyazikhin et al. 1998). However, in urban environments, the effect of surface pressure might be influenced by additional factors such as pollution and heat accumulation, which could offset some of its positive impacts. Future research could explore the interactions between air quality, atmospheric pressure, and tree health to refine our understanding of these complex relationships.

Moisture availability emerged as a critical factor for tree health, with a stronger effect in forests (β = 0.39, 95% CI: 0.29–0.49, p < 0.001) compared to urban areas (β = 0.30, 95% CI: 0.20–0.40, p < 0.001). This finding reinforces the importance of water availability for sustaining vegetation health, particularly during dry periods. The slightly weaker response in urban areas suggests that even when moisture levels are relatively high, trees in urban environments may struggle to access water due to impervious surfaces and altered hydrological processes (Brosinsky et al. 2013; Ugbaje and Bishop 2020). The implications for urban forestry management are clear: enhancing soil permeability, increasing green infrastructure, and implementing water conservation strategies can help improve moisture retention and support urban tree health. In forest ecosystems, maintaining natural hydrological cycles and preventing excessive soil drying will be crucial for sustaining tree productivity under changing climate conditions.

A central contribution of this study lies in its direct comparison of tree responses across urban and forest environments to climate variables. While the directional effects of climate factors were consistent across both environments, the magnitude of these effects differed significantly. Urban trees generally showed weaker positive responses to beneficial factors (leaf temperature, moisture) and stronger negative responses to stress factors (wind speed), highlighting the compounded challenges they face. This contrast underscores the heightened vulnerability of urban trees, which are exposed to more frequent and intense climate extremes compared to their forest counterparts. This reinforces the need for ecosystem-specific management strategies. Urban trees require interventions to mitigate heat stress, improve moisture access, and reduce wind exposure, whereas forest management should focus on maintaining natural climate resilience through conservation and sustainable water management. Moreover, managing urban green spaces to enhance their cooling effect through green infrastructure could significantly reduce the urban heat island effect, which is exacerbated by increasing temperatures.

This research establishes a robust statistical framework for analysing climate-vegetation interactions using satellite data, but several areas warrant further investigation. Future research should explore species-specific responses to climate variables, as different tree species may exhibit varying degrees of resilience to environmental stressors. Investigating how climate change interacts with urbanisation processes, such as urban heat islands and air pollution, would also provide a more nuanced understanding of the challenges faced by urban trees. Additionally, investigating lag effects between climate events and vegetation responses could offer deeper insights into how trees adapt to changing conditions over time.

Furthermore, integrating field-based physiological measurements with remote sensing data could enhance the accuracy of climate impact assessments on tree health. By incorporating on-the-ground monitoring, such as continuous leaf temperature measurements and chlorophyll content analysis (Lamontagne et al. 2000), this approach could help validate remote sensing data and provide a more comprehensive picture of tree health. This would allow researchers to capture fine-scale physiological processes that may not be fully represented by NDVI alone.

The findings of this study have significant implications for climate adaptation and urban forestry management. By identifying key climate drivers of tree health, policymakers and urban planners can develop targeted strategies to enhance tree resilience in both natural and built environments. In particular, the integration of climate data with spatial planning tools can help identify areas that would benefit most from increased greening efforts or strategic tree planting. Implementing adaptive management practices such as selecting heat-tolerant tree species, improving urban water retention, and designing wind-buffering landscapes can help mitigate the adverse effects of climate change on urban tree populations.

For forest management, strategies should focus on maintaining adequate soil moisture levels, preventing excessive canopy loss, and monitoring long-term climate trends to anticipate potential stressors. In addition, promoting biodiversity and ecosystem health through the conservation of native species and habitats will be critical in helping forests adapt to climate change. As climate change continues to reshape environmental conditions, proactive measures will be essential to ensure the long-term health and sustainability of tree ecosystems.

While this research draws on robust datasets and integrates physiological thresholds with remote sensing analysis, certain limitations must be acknowledged. These include the reliance on satellite-derived vegetation indices, which constrain species-specific interpretation, and the inability to fully disentangle mixed species responses in heterogeneous urban environments. Additionally, challenges related to the availability of ground-based data and the limitations of satellite imagery due to cloud cover and shadows, particularly in the UK, must be considered. These factors may limit the temporal resolution and accuracy of some findings. We have addressed these challenges through careful model selection and contextual interpretation, though future research would benefit from integrating ground-based physiological measurements and species-level data (Wang et al. 2021; Zhu et al. 2023).

It is critical to deepen our understanding of species-specific sensitivities, explore lagged vegetation responses to climatic stressors, and refine our integration of remote sensing with on-the-ground observations. As climate change continues to alter environmental baselines, proactive, evidence-led management will be essential. By tailoring interventions to the distinct dynamics of urban and forest ecosystems, we can better preserve the ecosystem services upon which communities increasingly depend.

## Conclusion

This study offers valuable insights into the complex interactions between climate variables and tree health within both forest and urban ecosystems. Between 2018 and 2023, we documented multiple heat stress periods, with leaf temperatures reaching up to 42.9 °C in the urban area of Milton Keynes. The findings revealed nuanced, ecosystem-specific responses to climatic drivers, highlighting the intricate mechanisms of vegetation adaptation in the face of climate extremes.

The research uncovered distinct patterns of tree health across different environments, with key climate variables playing pivotal roles. Leaf temperature was positively associated with vegetation health, although the magnitude of response differed significantly between forest and urban settings. Forest trees exhibited a stronger thermal response (β = 0.45, 95% CI: 0.35–0.55, p < 0.001) while urban trees showed a more dampened reaction (β = 0.32, 95% CI: 0.16–0.48, p < 0.001). These values reflect NDVI increases associated with a 3.82 °C rise in leaf temperature, offering ecologically meaningful insights into how trees respond to moderate heat. The heatwave events in 2019, 2020, and 2022 were particularly illuminating, underscoring the thermal stress variations experienced by different tree populations.

Wind speed and surface pressure emerged as critical secondary factors influencing tree health. Wind consistently demonstrated a negative impact on vegetation, with a more pronounced effect in urban areas **(**β = − 0.21, 95% CI: − 0.41 to − 0.02, p = 0.035) than in forests (β = − 0.17, 95% CI: − 0.29 to − 0.05, p = 0.004). Stable atmospheric conditions, indicated by surface pressure, supported vegetation resilience (urban: β = 0.17, 95% CI: 0.03–0.31, p = 0.02; forest: β = 0.14, 95% CI: 0.04–0.24, p = 0.005). Moisture availability proved to be a crucial determinant of tree survival, with forest trees demonstrating a stronger moisture response (β = 0.39, 95% CI: 0.29–0.49, p < 0.001) compared to their urban counterparts (β = 0.30, 95% CI: 0.20–0.40, p < 0.001).

The heatwave analysis revealed profound ecological implications, particularly for urban tree populations. Events exceeding 38 °C highlighted significant vulnerabilities, with the 2022 heatwave peaking at 42.9 °C illustrating the differential thermal tolerance between urban and forest ecosystems. The results highlight the pressing need for adaptive, ecosystem-specific management strategies.

Time-lag analysis revealed that vegetation responses to leaf temperature were not solely immediate (lag 0: β = 0.64, p < 0.001). A secondary, delayed effect was observed (lag + 1: β = 0.18, p < 0.001), suggesting that urban trees, in particular, may exhibit residual stress from prior-day thermal conditions. This highlights the need for management strategies that account for both immediate and short-term cumulative impacts.

From a policy perspective, the study supports the development of climate-resilient urban forestry practices, including the selection of heat-tolerant species, improvements in soil moisture retention, and urban design strategies that mitigate wind stress. In forested areas, strategies should include enhanced moisture monitoring, prevention of canopy loss, and broader climate adaptation planning.

While this study integrates physiological thresholds with remote sensing to assess vegetation stress, certain limitations remain. These include reduced species-specific resolution due to satellite-derived indices and challenges in interpreting mixed-species responses in heterogeneous urban areas. Limited ground-based data and issues such as cloud cover may also affect accuracy. Nonetheless, careful model selection and contextual interpretation helped mitigate these challenges. Future research should prioritise species-level data and integrate field-based physiological measurements to better understand lagged responses and support evidence-based ecosystem management under changing climatic conditions.

## Data Availability

All the data used for satellite data processing is available in the repository of GEE.

## References

[CR1] Ahrens CW, Challis A, Byrne M, Leigh A, Nicotra AB, Tissue D, Rymer P (2021) Repeated extreme heatwaves result in higher leaf thermal tolerances and greater safety margins. New Phytol 232(3):1212–1225. 10.1111/nph.17640 34292598 10.1111/nph.17640

[CR2] Allen CD, Breshears DD, McDowell NG (2015) On underestimation of global vulnerability to tree mortality and forest die-off from hotter drought in the Anthropocene. Ecosphere. 10.1890/ES15-00203.1

[CR3] Ambrose AR, Baxter WL, Wong CS, Burgess SSO, Williams CB, Næsborg RR, Koch GW, Dawson TE (2016) Hydraulic constraints modify optimal photosynthetic profiles in giant sequoia trees. Oecologia. 10.1007/s00442-016-3705-3 10.1007/s00442-016-3705-3 27553681

[CR4] Aram F, Higueras García E, Solgi E, Mansournia S (2019) Urban green space cooling effect in cities. Heliyon 5(4):e01339. 10.1016/j.heliyon.2019.e01339 31008380 10.1016/j.heliyon.2019.e01339 PMC6458494

[CR5] Ardila JP, Tolpekin VA, Bijker W, Stein A (2011) Markov-random-field-based super-resolution mapping for identification of urban trees in VHR images. ISPRS J Photogramm Remote Sens 66(6):762–775. 10.1016/j.isprsjprs.2011.08.002

[CR6] Armson D, Stringer P, Ennos AR (2012) The effect of tree shade and grass on surface and globe temperatures in an urban area. Urban for Urban Green. 10.1016/j.ufug.2012.05.002

[CR7] Baldeck CA, Asner GP, Martin RE, Anderson CB, Knapp DE, Kellner JR, Wright SJ (2015) Operational tree species mapping in a diverse tropical forest with airborne imaging spectroscopy. PLoS ONE 10(7):e0118403–e0118403. 10.1371/journal.pone.0118403 26153693 10.1371/journal.pone.0118403 PMC4496029

[CR8] Bates D, Mächler M, Bolker B, Walker S (2015) Fitting linear mixed-effects models using lme4. J Stat Softw 67(1):1–48. 10.18637/jss.v067.i01

[CR9] Berra EF, Gaulton R, Barr S (2019) Assessing spring phenology of a temperate woodland: a multiscale comparison of ground, unmanned aerial vehicle and Landsat satellite observations. Remote Sens Environ 223:229–242. 10.1016/j.rse.2019.01.010

[CR10] Bolker BM, Brooks ME, Clark CJ, Geange SW, Poulsen JR, Stevens MHH, White JSS (2009) Generalized linear mixed models: a practical guide for ecology and evolution. Trends Ecol Evol 24(3):127–135. 10.1016/j.tree.2008.10.008 19185386 10.1016/j.tree.2008.10.008

[CR11] Brockwell PJ, Davis RA (1991) Time series: theory and methods. Springer Series in Statistics. Springer. https://books.google.co.uk/books?id=_DcYu_EhVzUC

[CR12] Brosinsky A, Lausch A, Doktor D, Salbach C, Merbach I, Gwillym-Margianto S, Pause M (2013) Analysis of spectral vegetation signal characteristics as a function of soil moisture conditions using hyperspectral remote sensing. J Indian Soc Remote Sens 42:311–324. 10.1007/s12524-013-0298-8

[CR13] Çankaya S, Samet Eker SHA (2019) Comparison of least squares, ridge regression and principal component approaches in the presence of multicollinearity in regression analysis. Food Sci Technol 7:1166–1172. 10.24925/turjaf.v7i8.1166-1172.2515

[CR14] Cheela VRS, Michele John WB, Sarker P (2021) Combating urban heat island effect—a review of reflective pavements and tree shading strategies. Buildings. 10.3390/buildings11030093

[CR15] Chellemi DO, Britton KO (1992) Influence of canopy microclimate on incidence and severity of dogwood anthracnose. Botany 70:1093–1096. 10.1139/b92-134

[CR16] Cochavi A, Amer M, Stern R, Yakir D (2020) Using Sun-induced fluorescence and Carbonyl Sulfide flux to assess the response to seasonal heatwave in a citrus orchard. In: 22nd EGU General Assembly, Held Online 4-8 May, 2020, Id.8440. 10.5194/egusphere-egu2020-8440

[CR17] Dessai S, Lonsdale K, Lowe J, Harcourt R (eds) (2024) Quantifying climate risk and building resilience in the UK. Springer International Publishing, Cham. 10.1007/978-3-031-39729-5

[CR18] Dormann CF, Elith J, Bacher S, Buchmann C, Carl G, Carré G, Marquéz JRG, Gruber B, Lafourcade B, Leitão PJ, Münkemüller T, McClean C, Osborne PE, Reineking B, Schröder B, Skidmore AK, Zurell D, Lautenbach S (2013) Collinearity: a review of methods to deal with it and a simulation study evaluating their performance. Ecography 36(1):27–46. 10.1111/j.1600-0587.2012.07348.x

[CR19] ESA (2015) Sentinel-2 User Handbook, European Space Agency (ESA). https://sentinel.esa.int/documents/247904/685211/Sentinel-2_User_Handbook

[CR21] Feeley K, Martinez-Villa J, Perez T, Silva Duque A, Triviño Gonzalez D, Duque A (2020) The thermal tolerances, distributions, and performances of tropical montane tree species. Front for Global Change. 10.3389/ffgc.2020.00025

[CR22] Frank SD, Just MG (2020) Can cities activate sleeper species and predict future forest pests? A case study of scale insects. Insects 11:142. 10.3390/insects11030142 32106554 10.3390/insects11030142 PMC7142728

[CR23] Goñas M, Rojas-Briceño NB, Culqui-Gaslac C, Arce-Inga M, Marlo G, Pariente-Mondragón E, Oliva-Cruz M (2022) Carbon sequestration in fine aroma cocoa agroforestry systems in Amazonas, Peru. Sustainability 14(15), Article 15. 10.3390/su14159739

[CR24] Graham M (2003) Confronting multicollinearity in ecological multiple regression. Ecology 84:2809–2815. 10.1890/02-3114

[CR25] Harrison XA, Donaldson L, Correa-Cano ME, Evans J, Fisher DN, Goodwin CED, Robinson BS, Hodgson DJ, Inger R (2018) A brief introduction to mixed effects modelling and multi-model inference in ecology. PeerJ. 10.7717/peerj.4794 10.7717/peerj.4794 PMC5970551 29844961

[CR26] IPCC (2023) Synthesis report of the IPCC sixth assessment report (AR6). In: Zhai DKDP (ed)

[CR27] Gu Z, Chen J, Shi P et al (2007) Correlation analysis of Normalized Different Vegetation Index (NDVI) difference series and climate variables in the Xilingole steppe, China from 1983 to 1999. Front Biol China 2:218–228. 10.1007/s11515-007-0033-3

[CR28] Kendon M, McCarthy M, Jevrejeva S, Matthews A, Sparks T, Garforth J, Kennedy J (2022) State of the UK Climate 2021. Int J Climatol 42(S1):1–80. 10.1002/joc.7787

[CR29] Khan R, Gilani H (2021) Global drought monitoring with big geospatial datasets using Google Earth Engine. Environ Sci Pollut Res. 10.1007/s11356-020-12023-0 10.1007/s11356-020-12023-0 33394397

[CR30] Khan R, Wheeler P, Gowing D (2025b) Thermal tolerance and recovery dynamics of urban tree species Acer campestre (field maple) under heat and drought stress derived from chlorophyll fluorescence. Research Square. 10.21203/rs.3.rs-6155610/v1

[CR31] Khan R, Wheeler P, Gowing D (2025a) Photosynthetic lag responses to heat stress in Acer campestre: Comparative analysis of record-breaking summer 2022 and moderate summer 2023 conditions. 10.22541/au.174264845.54187856/v1

[CR32] Knyazikhin Y, Kranigk J, Myneni RB, Panfyorov O, Gravenhorst G (1998) Influence of small-scale structure on radiative transfer and photosynthesis in vegetation canopies. J Geophys Res. 10.1029/97JD03380

[CR33] Krause GH, Winter K, Krause B, Jahns P, García M, Aranda J, Virgo A (2010) High-temperature tolerance of a tropical tree, *Ficus**insipida*: methodological reassessment and climate change considerations. Funct Plant Biol 37(9):890–900. 10.1071/FP10034

[CR34] Laćan I, McBride JR (2008) Pest Vulnerability Matrix (PVM): a graphic model for assessing the interaction between tree species diversity and urban forest susceptibility to insects and diseases. Urban For Urban Green. 10.1016/j.ufug.2008.06.002

[CR35] Lamontagne M, Bigras FJ, Margolis HA (2000) Chlorophyll fluorescence and CO2 assimilation of black spruce seedlings following frost in different temperature and light conditions. Tree Physiol 20(4):249–255. 10.1093/treephys/20.4.249 12651461 10.1093/treephys/20.4.249

[CR36] Marando F, Salvatori E, Sebastiani A, Fusaro L, Manes F (2019) Regulating ecosystem services and green infrastructure: assessment of urban heat island effect mitigation in the municipality of Rome, Italy. Ecol Model. 10.1016/j.ecolmodel.2018.11.011

[CR37] Marchin RM, Esperon-Rodriguez M, Tjoelker MG, Ellsworth DS (2022) Crown dieback and mortality of urban trees linked to heatwaves during extreme drought. Sci Total Environ 850:157915. 10.1016/j.scitotenv.2022.157915 35944640 10.1016/j.scitotenv.2022.157915

[CR38] Masuda K, Yamada T, Kagawa Y, Fukuda H (2022) Application of time lags between light and temperature cycles for growth control based on the circadian clock of *Lactuca Sativa* L. seedlings. Front Plant Sci. https://www.paperdigest.org/paper/?paper_id=pubmed-36589103 10.3389/fpls.2022.994555 PMC9802636 36589103

[CR39] Moser-Reischl A, Uhl E, Rötzer T, Biber P, Con T, Tan NT, Pretzsch H (2018) Effects of the urban heat Island and climate change on the growth of Khaya senegalensis in Hanoi, Vietnam. For Ecosyst 5(1):1–14. 10.1186/S40663-018-0155-X/TABLES/7

[CR40] Murakami D, Peters GW, Matsui T, Yamagata Y (2021) Spatio-temporal analysis of urban heatwaves using tukey g-and-h random field models. IEEE Access 9:79869–79888. 10.1109/ACCESS.2020.3013255

[CR41] OS (2025) OS Download Products’ Documentation. https://docs.os.uk/os-downloads

[CR42] Patra S, Chakraborty D, Verma VK, Pande R, Sangma RHC, Chakraborty M, Layek J, Hazarika S (2024) Influence of shifting thermal regimes on tomato fruit borer, *Helicoverpa**armigera* (Hubner) in the Eastern Himalaya: implications for pest management strategies. Int J Biometeorol 68(11):2241–2251. 10.1007/s00484-024-02741-2 39136711 10.1007/s00484-024-02741-2

[CR43] Perez TM, Feeley KJ (2020) Photosynthetic heat tolerances and extreme leaf temperatures. Funct Ecol 34(11):2236–2245. 10.1111/1365-2435.13658

[CR44] Prudente VHR, Martins VS, Vieira DC, de França e Silva NR, Adami M, Sanches IDA (2020) Limitations of cloud cover for optical remote sensing of agricultural areas across South America. Remote Sens Appl: Soc Environ 20:100414. 10.1016/j.rsase.2020.100414

[CR45] Qasim M, Amin M, Sarwar M (2020) Effect of different biochemical traits on seed cotton yield: an application of LIU linear regression. J Anim Plant Sci. 10.36899/JAPS.2020.6.0174

[CR46] Rijnhart JJM, Twisk JWR, Valente MJ, Heymans MW (2022) Time lags and time interactions in mixed effects models impacted longitudinal mediation effect estimates. J Clin Epidemiol 151:143–150. 10.1016/j.jclinepi.2022.07.004 35961442 10.1016/j.jclinepi.2022.07.004

[CR47] Roger JC, Ray JP, Vermote EF (2020) MOD09GQ v061 MODIS/Terra Surface Reflectance Daily L2G Global 250 m SIN Grid. LSR Product User Guide. https://lpdaac.usgs.gov/products/mod09gqv006/

[CR48] Sabater M (2019) ERA5-Land monthly averaged data from 1950 to present. Copernicus Climate Change Service (C3S) Climate Data Store (CDS). 10.24381/cds.68d2bb30

[CR49] Schreiber U, Berry JA (1977) Heat-induced changes of chlorophyll fluorescence in intact leaves correlated with damage of the photosynthetic apparatus. Planta. 10.1007/BF00385990 10.1007/BF00385990 24420396

[CR50] Shrestha N (2020) detecting multicollinearity in regression analysis. Am J Appl Math Stat 8(2), Article 2. 10.12691/ajams-8-2-1

[CR51] Singh P, Singh SH, Paprzycki M (2023) Detection and elimination of multicollinearity in regression analysis. Int J Knowl Based Intell Eng Syst. 10.3233/KES-221622

[CR52] Stone B, Rodgers MO (2001) Urban form and thermal efficiency: how the design of cities influences the urban heat island effect. J Am Plann Assoc 67(2):186–198. 10.1080/01944360108976228

[CR53] Tadrist L, Saudreau M, Hémon P, Amandolese X, Marquier A, Leclercq T, Langre E (2018) Foliage motion under wind, from leaf flutter to branch buffeting. J R Soc Interface. 10.1098/rsif.2018.0010 10.1098/rsif.2018.0010 PMC6000177 29743271

[CR54] Tan X, Liao J, Bedra KB, Li J (2021) Evaluating the 3D cooling performances of different vegetation combinations in the urban area. J Asian Architect Build Eng. 10.1080/13467581.2021.1903905

[CR55] Ugbaje SU, Bishop TFA (2020) Hydrological control of vegetation greenness dynamics in Africa: a multivariate analysis using satellite observed soil moisture, terrestrial water storage and precipitation. Land. 10.3390/land9010015

[CR56] UKCEH (2022) The UKCEH Land Cover Maps for 2017, 2018 and 2019. https://www.ceh.ac.uk/ukceh-land-cover-maps

[CR57] Vatcheva KP, Lee M, McCormick JB, Rahbar MH (2016) Multicollinearity in regression analyses conducted in epidemiologic studies. Epidemiology 6(2):227. 10.4172/2161-1165.1000227 27274911 10.4172/2161-1165.1000227 PMC4888898

[CR58] Wang Y, Akbari H (2016) the effects of street tree planting on urban heat island mitigation in Montreal. Sustain Cities Soc. 10.1016/j.scs.2016.04.013

[CR59] Wang X, Dallimer M, Scott CE, Shi W, Gao J (2021) Tree species richness and diversity predicts the magnitude of urban heat island mitigation effects of greenspaces. Sci Total Environ. 10.1016/j.scitotenv.2021.145211 10.1016/j.scitotenv.2021.145211 33513510

[CR60] Wasko C, Shao Y, Vogel E, Wilson L, Wang QJ, Frost A, Donnelly C (2020) Understanding trends in hydrologic extremes across Australia. J Hydrol 593:125877. 10.1016/j.jhydrol.2020.125877

[CR61] Weather Spark (2025) Milton Keynes Climate, Weather By Month, Average Temperature (United Kingdom)—Weather Spark. https://weatherspark.com/y/45412/Average-Weather-in-Milton-Keynes-United-Kingdom-Year-Round

[CR62] Wei X, Zhang J (2019) Patterns and mechanisms of diminishing returns from beneficial mutations. Mol Biol Evol. 10.1093/molbev/msz035 10.1093/molbev/msz035 PMC6681633 30903691

[CR63] Wi SH, Lee HJ, An S, Kim SK (2020) Evaluating growth and photosynthesis of kimchi cabbage according to extreme weather conditions. Agronomy. 10.3390/agronomy10121846

[CR64] Willan AR, Watts DG (1978) Meaningful multicollinearity measures. Technometrics. 10.1080/00401706.1978.10489694

[CR65] Willis KS (2015) Remote sensing change detection for ecological monitoring in United States protected areas. Biol Cons 182:233–242. 10.1016/j.biocon.2014.12.006

[CR66] Zhang T, Xiao-Ping Zhou XFL (2020) Reliability analysis of slopes using the improved stochastic response surface methods with multicollinearity. Eng Geol. 10.1016/j.enggeo.2020.105617

[CR67] Zhu L, Scafaro AP, Vierling E, Ball MC, Posch BC, Stock F, Atkin OK (2023) Heat tolerance of a tropical-subtropical rainforest tree species Polyscias elegans: time-dependent dynamic responses of physiological thermostability and biochemistry. New Phytol. 10.1111/nph.19356 10.1111/nph.19356 37932881

